# Association of the polymorphism of the vitamin D receptor gene (*VDR*) with the risk of leprosy in the Brazilian Amazon

**DOI:** 10.1042/BSR20204102

**Published:** 2021-07-06

**Authors:** Jasna Letícia Pinto Paz, Maria do Perpétuo Socorro Corrêa Amador Silvestre, Letícia Siqueira Moura, Ismari Perini Furlaneto, Yan Corrêa Rodrigues, Karla Valéria Batista Lima, Luana Nepomuceno Gondim Costa Lima

**Affiliations:** 1 Ph.D. Program in Parasitic Biology in the Amazon Region (PPGBPA), State University of Pará (UEPA), Tv. Perebebuí, 2623 - Marco, Belém - PA, 66087-662 - Belém, Pará, Brazil; 2 Bacteriology and Mycology Section (SABMI), Evandro Chagas Institute (IEC), Ministry of Health, Rodovia BR-316 km 7 s/n - Levilândia - 67030-000 - Ananindeua, Pará, Brazil; 3 University Center of the State of Pará (CESUPA), Av. Alm. Barroso, Nº 3775 - Souza - 66613-903 - Belém, Pará, Brazil; 4 Ph.D. Program in Epidemiology and Health Surveillance (PPGEVS), Evandro Chagas Institute (IEC), Ministry of Health, Rodovia BR-316 km 7 s/n - Levilândia - 67030-000 - Ananindeua, Pará, Brazil

**Keywords:** genotype, Leprosy, vitamin D receptor

## Abstract

The transmission and evolution of leprosy depends on several aspects, including immunological and genetic factors of the host, as well as genetic factors of *Mycobacterium leprae*. The present study evaluated the association of single nucleotide polymorphisms (SNPs) on the FokI (rs2228570), TaqI (rs731236), ApaI (rs7975232) regions of the vitamin D receptor (*VDR*) gene with leprosy. A total of 405 individuals were evaluated, composed by groups of 100 multibacillary (MB) and 57 paucibacillary (PB) patients, and 248 healthy contacts. Blood samples were collected from patients and contacts. The genotyping was performed by sequencing of the interest regions. The alleles of the studied SNPs, and SNP FokI genotypes, were not associated with leprosy. For the SNP on TaqI region, the relationship between the tt genotype, and for the SNP ApaI, the AA genotype, revealed an association with susceptibility to MB form, while Aa genotype with protection. The extended genotypes AaTT and AaTt of ApaI and TaqI were associated with protection against MB form. Further studies analyzing the expression of the *VDR* gene and the correlation with its SNPs might help to clarify the role of polymorphisms on the immune response in leprosy.

## Introduction

Evolution, transmission and clinical spectrum of leprosy depend on host immunological and genetic factors and its etiological agent, *Mycobacterium leprae* [1,2].

The diverse clinical manifestations of leprosy include: Tuberculoid leprosy (TT) (HT), which is associated with an effective Th1 cellular immune response and host’s high resistance to infection by *M. leprae*, where formation of a well-defined granuloma and limited lesions are observed as the main clinical signs. At the other pole, the deficiency in the Th1 cellular immune response leads to the lepromatous leprosy (LL) (HV), a form of host’s high susceptibility to infection characterized by excessive bacillary multiplication and infection spread. Also, dimorphic forms of leprosy (DL) include clinical manifestations in between these polar forms, where hosts may present characteristics of TT and LL, or simply remain dimorphic (DL) [1–3].

In 2019, Brazil was the second most endemic country with the disease with 23612 new cases and a detection rate of 13.70/100000, with the Northern region being the third with 4599 new cases [4].

Several studies highlight that vitamin D deficiency increases the risk of infectious diseases such as leprosy and tuberculosis (TB), in addition to other conditions, including cancer, type 2 diabetes mellitus and cardiovascular and autoimmune diseases. Even before discovery of effective antibiotics, positive effects of vitamin D have been observed in leprosy treatment along with sulfones, and for TB in the form of cod liver oil and exposure to sunlight [5–7].

Vitamin D is nutritionally derived from a limited number of foods. The primary source is obtained from the conversion of 7-dehydrocholesterol in the skin, induced by exposure to the sun’s ultraviolet B rays (UVB). Vitamin D is converted in the liver into 25-hydroxyvitamin D3 (25-OHD3) and subsequently hydroxylated in the kidney by means of 1-α-hydroxylase to 1,25-dihydroxyvitamin D3 (1,25(OH)2D3) enzyme. 25-OHD3 is transported to organs, tissues and cells through vitamin D-binding protein (VDBP), which enters macrophages and converts into 1,25(OH)2D3 by the enzyme 1-α-hydroxylase and mitochondrial CP27B, finally binding to the vitamin D receptor (VDR) in the cell [7–9]. The 1,25(OH)2D3 once linked to VDR is activated and undergoes through a conformational change, inducing a superoxide burst, which enhances the fusion of phagolysosome in infected macrophages and the expression of genes involved in the synthesis of catelecidin (antimicrobial peptide), and in the differentiation of monocytes, macrophages, dendritic cells and neutrophils [7–9].

VDR is present in the nucleus of bone cells, kidneys, intestines, heart, skin, brain, liver, reproductive, endocrine and immune systems, acting as a regulator in the expression of genes related with cell growth, maturation, secretion of insulin, and modulation of the function of activated T and B lymphocytes, macrophages and production of cytokines. Within the innate immunity, it plays a major role in toll-like receptors (TLR) activation in monocytes, enhancing their microbicidal performance [10–12].

Deregulation of the vitamin D pathway can be influenced by single nucleotide polymorphisms (SNPs) in the *VDR* gene, that regulate transcriptional activity in response to intracellular pathogens, which may result in differential gene transcription, and consequently associated with changes in bone mineral density, calcium absorption, metabolic disorders and susceptibility to infectious diseases [11,13,14].

The main functional SNPs in the *VDR* gene are: the FokI T>C in position ch12: 47879112 (rs2228570, T allele designated ‘f’ and C allele designated ‘F’), TaqI T>C in position chr12: 47844974 (rs731236, T allele designated as ‘T’ and C allele designated as ‘t’) and ApaI G>T in position chr12: 47845054 (rs7975232, G allele designated as ‘a’ and T designated as ‘A’) [13,14].

Different populations have different genetic configurations of the *VDR* gene, which may reflect in different associations of SNPs with certain diseases [13,15]. Futhermore, studies involving the detection of *VDR* gene SNPs in leprosy patients and their healthy contacts in different populations may elucidate aspects of individuals’ immunogenic susceptibility to leprosy and disease severity. Therefore, the present study aimed to evaluate the association of SNPs FokI (rs2228570), TaqI (rs731236), ApaI (rs7975232) of the *VDR* gene with leprosy.

## Methods

### Sampling and ethics considerations

The present study included individuals from Rondom do Pará, Curianópolis, Goianésia and Redenção cities, state of Pará, Amazon Region, Brazil. Two distinct groups were evaluated: patients and contacts. Patients group included individuals with a clinical diagnosis of leprosy, in treatment or who had concluded treatment, and who at the time of diagnosis were classified as paucibacillary (PB) or multibacillary (MB), according to the Ministry of Health of Brazil. The contacts group consisted of individuals who lived with leprosy patients during the disease course and did not become ill. Sample size was calculated using the QUANTO software (www.biostats.usc.edu/software), aiming to reach a power of 80%.

All participants provided written informed consent and the study was approved by the municipal health authorities and the Research Ethics Committee of the Evandro Chagas Institute (Ministry of Health) (CAAE 48723115.1.0000.0019).

### DNA extraction and genotyping

Blood samples were collected from patients and contacts, from which DNA extractions were performed using the DNeasy Blood and Tissue kit (QIAGEN), following the manufacturer’s guidelines. For the typification of the polymorphisms in FokI (rs2228570), TaqI (rs731236) and ApaI (rs7975232) regions, fragments of 897, 824 and 959 bp, respectively, were amplified by polymerase chain reaction (PCR). PCRs were performed on Veriti Thermocycler (Applied Biosystems, Foster City, CA, U.S.A.), under the following conditions for all genes: initial denaturation at 95°C for 15 min, followed by 40 cycles of denaturation at 94°C for 30 s, annealing at 56°C for 1:30 min, extension at 72°C for 1 min and extension 70°C for 10 min.

The amplified products were subjected to electrophoresis in 2% agarose gel containing 3 µl of Sybr Safe and amplicons were visualized under ultraviolet light, followed by sequencing using Big Dye Terminator v3.1 chemistry on ABI Prism 3130 Genetic Analyzer (Applied Biosystems, Foster City, CA, U.S.A.). Obtained sequences were edited and analyzed using the BioEdit 8.0 software and on BLAST public database—National Center for Biotechnology Information (NCBI) website.

Primers used in PCR and sequence reactions were designed in the present study using the Primer3Plus program (Untergasser et al., [32] 2012), and based on the genomic region of the vitamin D receptor (1,25-dihydroxyvitamin D3) in *Homo sapiens*, deposited in GenBank with the reference NC_018923.2 (Table 1).

**Table 1:** Primers designed by the Primer3Plus program from the genomic region of the vitamin D receptor (1,25-dihydroxyvitamin D3) *Homo sapiens*, deposited in GenBank under the reference NG_008731,1

SNP	Primer	pb
FokI (rs2228570)	FokI F: 5′-GCCAGCTATGTAGGGCGAAT-3′	897
	FokI R: 5′-TGACACTGCCTGGATGTTCC-3′	
ApaI (rs7975232)	ApaI F: 5′-GCCAAACACTTCGAGCACAA-3′	824
	ApaI R: 5′-TCCTCTCCCATGAAGCTTAGGA-3′	
TaqI (rs731236)	TaqI F: 5′-GCCAAACACTTCGAGCACAA-3′	959
	TaqI R: 5′-GAGATGGGCTCCTCTACCCC-3′	

Abbreviation: pb, fragment size in base pair.

### Statistical analysis

The G-test of independence, Chi-square (χ^2^) and/or Fisher’s exact test were applied to verify the association of polymorphisms presence within each studied group. Odds ratio (OR) were calculated to assess the association between exposure and the outcomes of interest. For FokI, TaqI and ApaI SNPs, the wild alleles ‘f’, ‘T’ and ‘a’, respectively, were considered risk or exposure factors.

Genotype frequencies of the control groups were tested based on the Hardy–Weinberg balance associated with the Chi-square test (χ^2^). Values of *P*≤0.05 were considered statistically significant and all analyses were performed using the statistical software, GraphPad Prism version 8.00.

## Results

The genotypic frequencies in the study population agreed with the Hardy–Weinberg equilibrium (*P*>0.05), using the chi-square test (χ^2^) for the TaqI (*P*=0.56) and ApaI (*P*=0.74) and FokI (*P*=0.62).

Among all individuals, SNP analysis for FokI rs2228570 region revealed a higher prevalence of the Ff (49.3%) genotype when compared with the ff (13.2%) genotype. No statistically significant differences between the presence of specific genotype in the studied groups and risk association between the SNP genotype and leprosy were observed (Tables 2 and 3).

**Table 2:** Distribution of SNP genotypes FokI rs2228570, TaqI rs731236, ApaI rs7975232 in the studied groups

Groups	FokI				TaqI				ApaI			
	FF	Ff	ff	*P*	TT	Tt	tt	*P*	AA	Aa	aa	*P*
MB	18 (46.2%)	18 (46.2%)	03 (7.6%)	0.67	43 (55.8%)	23 (29.9%)	11 (14.3%)	0.26	34 (42.0%)	26 (32.1%)	21 (25.9%)	<0.00^*^
PB	08 (42.1%)	08 (42.1%)	03 (15.8%)		23 (57.5%)	15 (37.5%)	02 (5.0%)		12 (27.9%)	27 (62.8%)	04 (9.3%)	
Groups												
MB	18 (46.2%)	18 (46.2%)	03 (7.6%)	0.28	43 (55.8%)	23 (29.9%)	11 (14.3%)	0.07	34 (42.0%)	26 (32.1%)	21 (25.9%)	<0.00^*^
PB+Contacts	36 (34.0%)	54 (50.9%)	16 (15.1%)		154 (57.3%)	99 (36.8%)	16 (5.9%)		84 (30.9%)	140 (51.5%)	48(17.6%)	
**Groups**												
Patients	26 (44.8%)	26 (44.8%)	06 (10.4%)	0.29	66 (56.4%)	38 (32.5%)	13 (11.1%)	0.26	46 (37.1%)	53 (42.7%)	25 (20.2%)	0.46
Contacts	28 (32.2%)	46 (52.9%)	13 (14.9%)		131 (57.2%)	84 (36.7%)	14 (6.1%)		72 (31.4%)	113 (49.4%)	44 (19.2%)	
**Groups**												
PB	08 (42.1%)	08 (42.1%)	03 (15.8%)	0.68	23 (57.5%)	15 (37.5%)	02 (5.0%)	0.96	12 (27.9%)	27 (62.8%)	04 (9.3%)	0.18
Contacts	28 (32.2%)	46 (52.9%)	13 (14.9%)		131 (57.2%)	84 (36.7%)	14 (6.1%)		72 (31.4%)	113 (49.4%)	44 (19.2%)	
**Groups**												
MB	18 (46.2%)	18 (46.2%)	03 (7.6%)	0.25	43 (55.8%)	23 (29.9%)	11 (14.3%)	0.09	34 (42.0%)	26 (32.1%)	21 (25.9%)	0.03^*^
Contacts	28 (32.2%)	46 (52.9%)	13 (14.9%)		131 (57.2%)	84 (36.7%)	14 (6.1%)		72 (31.4%)	113 (49.4%)	44 (19.2%)	

Chi-square or G-test of independence (Chi-square residue analysis), as needed.

*Statistically significant.

**Table 3:** Risk associations among the SNP genotypes FokI rs2228570, TaqI rs731236, ApaI rs7975232 and the groups

Genotypes FokI	Patients, *n* (%)	Con *n* (%)	OR [CI 95%]	*P*	MB, *n* (%)	PB, *n* (%)	OR [CI 95%]	*P*	MB, *n* (%)	PB+Con, *n* (%)	OR [CI 95%]	*P*
**Ff**	26 (44.8%)	46 (52.9%)	1.225 [0.39–3.71]	0.79	18 (46.2%)	8 (42.1%)	2.250 [0.43–11.06]	0.38	18 (46.2%)	54 (50.9%)	1.778 [0.49–6.25]	>1.00
**FF**	26 (44.8%)	28 (32.2%)	2.012 [0.71–6.34]	0.28	18 (46.2%)	8 (42.1%)	2.250 [0.43–11.06]	0.39	18 (46.2%)	36 (34.0%)	2.667 [0.71–9.41]	>1.00
**Ff e FF**	52 (89.6%)	74 (85.1%)	1.523 [0.55–4.25]	0.46	36 (92.4%)	16 (84%)	2.250 [0.47–10.31]	0.38	36 (92.4%)	90 (84.9%)	2.133 [0.59–7.21]	>1.00
**ff (wild)**	6 (10.4%)	13 (14.9%)	1 [-]	-	3 (7.6%)	3 (15.8%)	1 [-]	-	3 (7.6%)	16 (15.1%)	1 [-]	>1.00
**TaqI**												
**tt**	13 (11.1%)	14 (6.1%)	1.843 [0.81–4.05]	>1.00	11 (14.3%)	02 (5.0%)	2.942 [0.70–14.06]	0.21	11 (14.3%)	16 (5.9%)	2.462 [1.11–5.50^*^]	0.05*
**Tt**	38 (32.5%)	84 (36.7%)	0.898 [0.56–1.44]	>1.00	23 (29.9%)	15 (37.5%)	0.820 [0.37–1.86]	0.67	23 (29.9%)	99 (36.8%)	0.832 [0.46–1.45]	0.57
**tt e Tt**	51 (43.6%)	98 (42.8%)	1.033 [0.65–6.11]	>1.00	34 (44.2%)	17 (42.5%)	1.070 [0.49–2.31]	1.00	34 (44.2%)	115 (42.7%)	1.059 [0.62–1.75]	0.89
**TT (wild)**	66 (56.4%)	131 (57.2%)	1 [-]	>1.00	43 (55.8%)	23 (57.5%)	1 [-]	-	43 (55.8%)	154 (57.3%)	1 [-]	
** ApaI**												
**Aa**	53 (42.7%)	113 (49.4%)	0.82 [0.46–1.46]	>1.00	26 (32.1%)	27 (62.8%)	0.183 [0.06–0.61^*^]	0.00*	26 (32.1%)	140 (51.5%)	0.425 [0.22–0.81^*^]	0.01*
**AA**	46 (37.1%)	72 (31.4%)	1.124 [0.60–2.07]	>1.00	34 (42.0%)	12 (27.9%)	0.54 [0.17–1.87]	0.39	34 (42.0%)	84 (30.9%)	0.92 [0.48–1.74]	0.87
**Aa e A/A**	99 (79.8%)	185 (80.8%)	0.94 [0.55–1.61]	>1.00	60 (74.1%)	39 (90.7%)	0.29 [0.10–0.85^*^]	0.03	60 (74.1%)	224 (82.4%)	0.612 [0.34–1.09]	0.11
**aa (wild)**	25 (20.2%)	44 (19.2%)	1 [-]	>1.00	21 (25.9%)	4 (9.3%)	1 [-]	-	21 (25.9%)	48 (17.6%)	1 [-]	-

Abbreviations: BCG, Bacillus Calmette-Guérin vaccine; CI 95%, confidence interval; Con, contacts. *Statistically significant.

The TT genotype was the most prevalent (56.9%) when compared with the tt genotype (7.8%) on the SNP analysis of TaqI rs731236 region among all evaluated individuals. Also, no statistically significant differences between the presence of specific genotype in the studied groups was observed (Table 2). The presence of the mutant tt genotype was associated with the MB leprosy manifestation, with chance of progression to this disease form increased twice (OR = 2.46; confidence interval (CI 95%) = 1.11–5.51) when compared with PB form and contacts (Table 3), and when only the MB and PB forms of leprosy were compared (OR = 2.9; CI 95% = 1.4–5.2) (Table 4).

**Table 4:** Risk analyses of the extended SNPs ApaI and TaqI genotypes between MBs and the union of PBs and contacts

APAI/TAQI	MB	PB+Contacts	OR	*P-*value	CI 95%
**AaTT**	15	81	0.41	0.02	0.19–0.94^*^
**AaTt**	08	57	0.31	0.01	0.12–0.81^*^
**aaTt**	01	04	0.55	1	0.04–3.91
**AATT**	10	31	0.72	0.5	0.27–1.85
**AATt**	12	37	0.72	0.5	0.31–1.72
**aatt**	01	02	1.11	1	0.07–10.10
**AAtt**	10	14	1.59	0.4	0.62–4.45
**aaTT (Wild)**	17	38	1	-	-

*Statistically significant.

SNP analysis for ApaI rs7975232 region demonstrated a high prevalence of the Aa (47.03%) genotype, followed by aa genotype (19.55%). The presence of AA genotype was associated with MB patients, while Aa genotype was significantly higher among PB patients (*P*<0.00; Table 2), and in the group incluiding PB patients and contacts (*P*=0.0088; Table 2). In addition, when considering only MB patients and contacts, the Aa genotype was detected in significantly higher frequency among contacts (*P*<0.00). Risk analysis revealed that Aa genotype was associated with less chance of MB manifestation of leprosy when compared with the group comprising the PB patients and contacts (OR = 0.42; CI 95% = 0.22–0.81; Table 3), and when compared only between MB and PB patients (OR = 0.18; CI 95% = 0.063–0.61).

Regarding the analysis of the extended SNPs genotypes for the TaqI rs731236 and ApaI rs7975232 regions, patients presenting the AaTT and AaTt genotypes were 60 and 70% less susceptible to clinically manifest the MB form of leprosy when compared with the group comprising the PB patients and contacts (OR = 0.1; CI 95% = 0.9–0.5; OR = 0.1; CI 95% = 0.12–0.81, respectively; Table 4).

In the comparisons including the wild haplotype using alleles frequencies, and considering the chance of presenting the worst outcome of the disease (MB) instead of developing the PB form together with the resistance (through the contacts), it was possible to observe that the a-t-f haplotype was associated with an approximately 90% lower chance of this outcome (OR = 0.1; 95% CI = 0.03–0.31; Table 5).

**Table 5:** Haplotype analysis using the allele frequencies of the investigated SNPs between MBs and the union of PCs and contacts

Haplotypes APAI/TAQI/FOKI	MB	PB+Contacts	*P*-value	OR [CI 95%]
ATF	81	296	0.3	0.7 [0.43–1.31]
Atf	16	46	1	0.96 [0.45–2.04]
AtF	46	104	0.5	1.2 [0.66–2.24]
atF	37	92	0.7	1.11 [0.59–2.08]
atf	4	108	<0.00	0.1 [0.03–0.31]
aTF	54	132	0.7	1.12 [0.62–2.04]
aTf (Wild)	21	58	-	-

Finally, no significant differences were observed in the presence of a specific allele of the genes and evaluated groups, as well as of the presence of a specific allele and chance of a given outcome (Table 6).

**Table 6:** Distribution of the frequency of FokI, TaqI and ApaI polymorphism alleles by group of individuals investigated

Alleles FokI	Patients, *n* (%)	Contacts, *n* (%)	OR [CI 95%]	MB, *n* (%)	PB, *n* (%)	OR [CI 95%]	MB, *n* (%)	PB+Contacts, *n* (%)	OR [CI 95%]
**F**	78 (67.2%)	101 (58.7%)	1.313 [0.5776–2.870]	54 (69.2%)	24 (63.2%)	1.313 [0.5776–2.870]	54 (69.2%)	125 (59.5%)	1.530 [0.897 –2.674]
**f**	38 (32.8%)	71 (41.3%)		24 (30.8%)	14 (36.8%)		24 (30.8%)	85 (40.5%)	
**Alleles TaqI**									
**T**	170 (72.65%)	346 (75.6%)	1.163 [0.811–1.667]	109 (70.8%)	61 (76.3%)	1.325 [0.714–2.404]	109 (70.8%)	407 (75.6%)	1.283 [0.866–1.911]
**T**	64 (27.35%)	112 (24.4%)		45 (29.2%)	19 (23.7%)		45 (29.2%)	131 (24.4%)	
**Alleles ApaI**									
**A**	145 (58.47%)	257 (56.11%)	1.101 [0.803–1.500]	94 (58.0%)	51 (59.3%)	0.949 [0.560–1.636]	94 (58.0%)	308 (56.62%)	1.059 [0.741–1.506]
**A**	103 (41.53%)	201 (43.89%)		68 (42.0%)	35 (40.7%)		68 (42.0%)	236 (43.38%)	

*P*>0.05.

## Discussion

The individual mechanisms related with leprosy and its progression to a determined clinical manifestation still remain to be deciphered. Immunogenetic studies on the host’s defense mechanisms and mapping of human genome for leprosy suspectibility (or protection) markers represent a powerful tool for early and more complex prophylactic interventions among a determined risk group.

The participation of vitamin D in immunological processes of defense against mycobacterial infections is well reported. In order to perform its function, vitamin D requires its receptors’ proper production activation. VDR is a nuclear protein, composed of 437 amino acids, encoded by the *VDR* gene located on chromosome 12 and composed of 11 exons. When VDR binds to vitamin D, it is activated and binds to the VDR response element, regulating the transcription of different classes of genes involved in the host immune response. Thus, VDR expression levels are important in controlling disease progression. VDR also plays a major role in miRNA regulation, and consequently, indirectly involved in other genes regulation [17,18]. Futhermore, the expression of the *VDR* gene among leprosy patients is approx. 5–10% lower when compared with healthy individuals, also reflection in lower levels of 1,25(OH)2D3 in the blood of patients [17]. Thus, changes in the 5′ region on the *VDR* gene promoter can alter expression patterns and mRNA levels, while variations on the 3′ region (RTU) may affect mRNA stability and efficiency of protein translation [11,15,16].

SNP analysis for the FokI rs2228570 region revealed no significant association with leprosy, in line with previous report in India by Sapkota et al. [24] (2010). However, two other studies conducted in India associated the ff genotype with susceptibility to leprosy [11,17]. This last genotype was also associated with a 34% higher risk for TB in a meta-analysis study, which analyzed 32 case–control studies. In another report, the ff genotype was associated with a high risk of multidrug-resistant (MDR) TB and high load of bacilli in positive smear microscopy. In addition, the f allele was also associated with multiple sclerosis [18–20].

Our data revealed that the FF genotype was present in 37.5% of the individuals and ff was the less frequent (13.2%). The substituion of the T nucleotide (f) to C nucleotide (F) in the FokI region located in exon 2 produces a 424-amino acid truncated protein. Thus, the F allele codifies a shorter variant protein, which enhances binding and role of vitamin D. Individuals presenting the FF genotype are expected to have less susceptibility, while the ff genotype is related with a higher susceptibility to leprosy [13,14].

Considering the SNP analysis for the TaqI rs731236 region, the relationship between the tt genotype and MB presentation of leprosy was observed, as individuals with this genotype had twice higher chances of progression to the MB form. This genotype was associated with leprosy in India, Africa and Southeastern Brazil, and also associated with TB in African populations [11,15,21–24]. In Southeastern Brazil, researchers demonstrated a 13-times greater risk of developing leprosy in individuals with this genotype combined with a negative Mitsuda test [22]. In TB, the tt genotype has been associated with the development of MDR and increased bacillary load on sputum smear microscopy. Interestingly, a study conducted in Mexico revealed that the genotype associated with the MB form was TT, and Sapkota et al. (2010) and Neela et al. (2015) also found no relationship between SNP rs731236 and leprosy [15,17,18,24]. Recently, a study in southern Brazil demonstrated no association between this SNP and leprosy, but associated the bAt haplotype formed by the SNPs in BsmI, ApaI and TaqI genes with leprosy *per se* [13].

The TaqI rs731236 region is located in exon 9, close to the 3′UTR (untranslated region) of the *VDR* gene, and the exchange of T nucleotide (T) with C nucleotide (t) does not change the amino acid (isoleucine) for this codon, therefore, not altering the structure of the VDR protein. Although it is reported that the mRNA for the T allele is less stable than the mRNA for the t allele, the association of this allele with the quantitative expression of the VDR mRNA has not yet been confirmed, and we suggest that the tt genotype favors susceptibility to leprosy. According to Goulart et al. (2009), the tt genotype probably affects the T cells differentiation and maturation, preventing an effective response of the cellular immune system and leading to deficiency on immunity mediated by cells [6,11,15,22,23].

The SNP analysis for the ApaI rs7975232 region demonstrated that the AA genotype was associated with susceptibility to the MB form, while the Aa genotype as a protection marker (present among PB form patients and contacts). The Aa genotype was also associated with a 60% lower chance of individuals to develop the MB manifestations, suggesting a 40% protection estimate for individuals with this genotype. Nevertheless, in report by Neela et al. [17] (2015) these two genotypes were associated with leprosy *per se*, demonstrating the association of the allele A with leprosy, and two studies carried out in Africa and in Southeastern Brazil, found no relationship between this SNP and leprosy. In TB, two studies associated the AA genotype with the resistant lung form, while a meta-analysis including six studies in a random effects model did not show any association between this SNP and disease. Also, this genotype was associated with Crohn’s disease [6,25–27]. Also, the SNP on the ApaI rs7975232 regions is located at intron 9, close to the 3′UTR region of the *VDR* gene, and has been observed that the substitution of G nucleotide (a) to T nucleotide (A) does not modify the VDR protein [28]. So far, an association between this SNP and the stability and/or expression of the VDR mRNA has not been confirmed.

Differently from what is observed in the allelic variant of exon 2 (FokI rs2228570), the mutations located at the 3′ end are in disequilibrium, and the extent and effects of this umbalance are distinct among the different ethnic groups, where previous reports highlight that ApaI and TaqI have a strong link of unbalance. [29]. Our data revealed that the association of the extended genotype for TaqI rs731236 and ApaI rs7975232 regions suggests that the genotypes AaTT and AaTt were associated with protection against MB form, and associated with a 60 and 70% lower chance of the individual evolve to the MB form, respectively. These results corroborate with the literature, in which the extended BBAAtt genotype was associated with maximum suppression in lymphocytes proliferation, leading to a possible ineffective cellular response and enhancing the susceptibility to leprosy [6,30,31].

When analyzing the haplotype using the frequency of the alleles, the a-t-f haplotype was associated with protection from the disease, in which the individual with this haplotype has a 90% lower chance of presenting the MB form. In another study, the t-F-a haplotype was associated with leprosy *per se* [11].

The influence of SNPs on the development of leprosy is likely to be exacerbated when associated with other genetic factors. Even though, each of these factors may independently contribute to evolution of leprosy, and the simultaneous occurrence of unfavorable factors is a predisposition factor for the disease. The polymorphisms evaluated in the present study have been previously analyzed and related in other diseases’ settings. However, data from several studies shows divergent results on associations of these SNPs with various diseases, where this divergence might explained by ethnic and genetic differences among the different populations evaluated; the influence that alleles of other genes may influence on the pathogenesis of the studied disease, and external factors such as diets and stress may affect gene expression.

Polymorphisms may also not specifically interfere in disease susceptibility, but on the ability to respond to bioavailable forms of vitamin D, highliting the importance of conduct studies in different populations, in addition to investigating other genomic regions, aiming to clarify the involvement of SNPs on the VDR gene and immune response triggered by pathogen-host contact. Also, the evaluating the extended genotype and haplotype of susceptibility to leprosy, which may still vary according to the ethnicity of the population studied. The assembly of an extended genotype and susceptibility haplotype in a given population may be extremely important indicator of higher susceptible to leprosy among individuals in a given region and, in the case of patient's contacts the susceptibility haplotype. Increment in monitoring and more effective and complex prophylaxis can be performed, such as the use of rifampicin and BCG vaccination, however, these measeures are more expensive and unlikely feasible to administer in all contacts. Thus, the present study contributes with data on the genotypes that may be present in futures population of Northern Brazil.

## Conclusion

No association of alleles of the studied SNPs and SNP of FokI genotypes with leprosy was observed. For the SNP on TaqI region, the relationship between the tt genotype, and for the SNP on ApaI, the AA genotype, were associated with susceptibility to MB form, while Aa genotype with protection. The extended genotypes AaTT and AaTt of ApaI and TaqI regions were associated with protection against MB form. The restricted number of analyzed samples may have contributed to the lack of statistical significance in data analyses. Future studies evaluating the expression of the *VDR* gene, and the correlation with its SNPs may explain the role of polymorphisms and its influence the immune response in leprosy.

## Data Availability

The authors confirm that all original raw data are available at the time of submission. As per the Data Policy, these data will be stored for a minimum of 10 years and will be made available to the Editorial Office, Editors and readers upon request.

## Abbreviations

CI 95%: confidence interval
MB: multibacillary
MDR: multidrug-resistant
OR: odds ratio
PB: paucibacillary
PCR: polymerase chain reaction
SNP: single nucleotide polymorphism
TB: tuberculosis
VDR: vitamin D receptor
25-OHD3: 25-hydroxyvitamin D3
1,25(OH)2D3: 1,25-dihydroxyvitamin D3
